# Supplementary material for: Spine and lower body symmetry during treadmill walking in healthy individuals—In-vivo 3-dimensional kinematic analysis
Source: PLoS One. 2022 Oct 6;17(10):e0275174. doi: 10.1371/journal.pone.0275174 (PMC9536630; doi:10.1371/journal.pone.0275174)
Supplement: Attachment — Submitted filename: Response to Reviewers_PLoS_One_Gait Symmetry TW.docx [file pone.0275174.s002.docx]

**AUTHOR’S RESPONSE TO REVIEWER’S COMMENTS	9/6/2022**

The authors wish to thank the Editor and Reviewers for their invaluable suggestions. The manuscript has been revised according to the editor’s and reviewers’ comments. A point-by-point reply is given below. The line, figure and table numbers noted in the replies refer to the revised manuscript. In the following response, the editor’s and reviewer’s comments are shown in italics and our responses appear immediately below in normal type.

**Editor:**

*Additional Editor Comments:*

*I have now received reviewers' comments from the required number of reviewers. You will see from the reviewers' comments that there are some minor outstanding matters that need to be addressed, before the manuscript can be accepted for publication; mostly around clarification of terms and concepts. I encourage you to consider and address them carefully in your reply, as per the instructions below.*

Reply: We thank the Editor for the comments. All comments have been addressed accordingly to increase the clarity for readership.

**Reviewer #3**

*Reviewer #3: This is an interesting paper on spinal and lower body symmetry during treadmill walking. The authors have addressed all previous comments and the paper flows significantly better. Large sections of the results and discussion section have been revised in order to increase clarity for readership.*

Reply: We thank the Reviewer for the comments.

**Reviewer #4**

*Reviewer #4: Title: Spine and lower body symmetry during treadmill walking in healthy individuals – in-vivo 3-dimensional kinematic analysis*

*The manuscript describes a cross-sectional observational study investigating the bilateral symmetry of 3-dimensional motions of the spine (four segments), pelvis, and lower limb joints during treadmill walking at an individual’s regular walking speed and fast walking speed (i.e., “as if they were in a hurry”). Overall, the manuscript is relatively well-written and clear, although a number of minor grammatical errors were noted in the document. Below are substantive comments for the authors to consider in their revisions.*

Reply: We thank the Reviewer for the comments. We have addressed all comments accordingly to increase the clarity for readership.

*Terminology*

*The terminology used to describe the 3-dimensional motions of the spine and pelvis segments are confusing.*

*Regarding spinal motion:*

*- “flexion/extension” is typically used for sagittal plane motion (rather than “posterior-anterior tilt”)*

*- “lateral flexion” is typically used for frontal plane motion (rather than “lateral tilt”)*

*- “(left/right) rotation” is typically used for transverse plane motion (rather than “lateral rotation”)*

*Regarding pelvis motion:*

*- “obliquity” is typically used for frontal plane motion (rather than “lateral tilt”)*

*- “(left/right) rotation” is typically used for transverse plane motion (rather than “lateral rotation”)*

*As well, the terms “ipsi-contra” or “contra-ipsi” are frequently used along with the motion terms – e.g., lower lumbar contra-ipsi lateral rotation (Line 38), upper lumbar ipsi-contra lateral tilt (Line 40). It is unclear what these represent – e.g., does “lower lumbar contra-ipsi lateral rotation” mean transverse plane rotation of the lower lumbar segment from the side contralateral to the leg being analyzed to the side ipsilateral to that leg?*

*It would be very helpful to use the ISB’s current guidelines regarding the terminology to use when describing the three-dimensional motions of the spine and pelvis segments to avoid confusion for the readers of this manuscript. Such changes would need to be made throughout the manuscript (e.g., Abstract, Results, Discussion, Tables, Figures).*

Reply: We agree and thank the Reviewer for the comments. The terminology has been revised throughout all Sections, as well as Figures and Tables of the manuscript to increase the clarity for readership.

**Line 38-41**:

*The lower lumbar left-right rotation (5.74±0.04°) and hip internal rotation (5.33±0.18°) presented the largest degrees of asymmetry during normal treadmill. Upper lumbar left-right lateral flexion (1.48±0.14°) and knee flexion (2.98±0.13°) indicated the largest asymmetries and during fast treadmill walking.*

*Figure 3.*

*Figure 4.*

*Figure 5.*

*Figure 6.*

**Table 1.** Average and standard deviation of the degree of asymmetry and maximum magnitude of the normalized symmetry index between the associated left and right motions in spine segments during normal and fast treadmill walking of N=60 participants.

| Segment | Treadmill Walking Normal | | | |  | Treadmill Walking Fast | | | |
| --- | --- | --- | --- | --- | --- | --- | --- | --- | --- |
| *Upper Lumbar* | *Flexion-Extension* | | |  |  | *Flexion-Extension* | | |  |
|  | *Gait Cycle %* | *DoA* | *p-value* | *max SI_norm_ %* |  | *Gait Cycle %* | *DoA* | *p-value* | *max SI_norm_ %* |
|  | 45 to 52 | 0.46±0.03 | 0.003 | 30 |  |  |  |  |  |
|  | 93 to 99 | 0.48±0.05 | 0.004 | 30 |  |  |  |  |  |
|  |  |  |  |  |  |  |  |  |  |
|  | *Left-Right Lateral Flexion* | | |  |  | *Left-Right Lateral Flexion* | | |  |
|  | *Gait Cycle %* | *DoA* | *p-value* | *max SI_norm_ %* |  | *Gait Cycle %* | *DoA* | *p-value* | *max SI_norm_ %* |
|  | 0 to 100 | 1.54±0.11 | <0.001 | 13 |  | 0 to 100 | 1.48±0.14 | <0.001 | 15.72 |
|  |  |  |  |  |  |  |  |  |  |
|  | *Left-Right Rotation* | | |  |  | *Left-Right Rotation* | | |  |
|  | Gait Cycle % | *DoA* | *p-value* | *max SI_norm_ %* |  | *Gait Cycle %* | *DoA* | *p-value* | *max SI_norm_ %* |
|  |  |  |  |  |  | 13 to 20 | 0.64±0.016 | 0.04 | 11 |
|  |  |  |  |  |  | 60 to 73 | 0.68±0.054 | 0.021 | 13.3 |
|  |  |  |  |  |  |  |  |  |  |
| *Lower Lumbar* | *Flexion-Extension* | | |  |  | *Flexion-Extension* | | |  |
|  | *Gait Cycle %* | *DoA* | *p-value* | *max SI_norm_ %* |  | *Gait Cycle %* | *DoA* | *p-value* | *max SI_norm_ %* |
|  | 2 to 3 | 0.48±0.01 | 0.049 | 29 |  | 9 to 19 | 0.68±0.05 | 0.001 | 30 |
|  | 4 to 16 | 0.45±0.09 | 0.002 | 32.7 |  | 58 to 68 | 0.51±0.12 | 0.001 | 34 |
|  | 55 to 66 | 0.42±0.09 | 0.005 | 32.7 |  |  |  |  |  |
|  |  |  |  |  |  |  |  |  |  |
|  | *Left-Right Lateral Flexion* | | |  |  | *Left-Right Lateral Flexion* | |  |  |
|  | *Gait Cycle %* | *DoA* | *p-value* | *max SI_norm_ %* |  | *Gait Cycle %* | *DoA* | *p-value* | *max SI_norm_ %* |
|  | 1 to 8 | 1.3±0.02 | 0.048 | 9.7 |  |  |  |  |  |
|  | 14 to 45 | 1.42±0.06 | 0.023 | 14 |  |  |  |  |  |
|  | 60 to 98 | 1.42±0.08 | 0.016 | 12.5 |  |  |  |  |  |
|  | 99 to 100 | 1.32±0.03 | 0.05 | 10 |  |  |  |  |  |
|  |  |  |  |  |  |  |  |  |  |
|  | *Left-Right Rotation* | | |  |  | *Left-Right Rotation* | | |  |
|  | *Gait Cycle %* | *DoA* | *p-value* | *max SI_norm_ %* |  | *Gait Cycle %* | *DoA* | *p-value* | *max SI_norm_ %* |
|  | 0 to 100 | 5.74±0.04 | 0.006 | 14.73 |  |  |  |  |  |

Abbreviations: DoA, degree of asymmetry; SI_norm_, normalized symmetry index.

**Table 2.** Average and standard deviation of the degree of asymmetry and maximum magnitude of the normalized symmetry index between the associated left and right motions in the pelvis segment and the lower body joints during normal and fast treadmill walking of N=60 participants.

| Segment | Treadmill Walking Normal | | | |  | Treadmill Walking Fast | | | |
| --- | --- | --- | --- | --- | --- | --- | --- | --- | --- |
| *Pelvis* | *Posterior-Anterior Tilt* | |  |  |  | *Posterior-Anterior Tilt* | | |  |
|  | *Gait Cycle %* | *DoA* | *p-value* | *max SI_norm_ %* |  | *Gait Cycle %* | *DoA* | *p-value* | *max SI_norm_ %* |
|  | 30 to 40 | 0.22±0.01 | 0.013 | 26.8 |  | 0 to 13 | 051±0.03 | 0.004 | 35.8 |
|  | 48 to 49 | 0.26±0.01 | 0.05 | 27.6 |  | 36 to 63 | 0.46±0.03 | <0.001 | 37.4 |
|  | 76 to 93 | 0.22±0.02 | 0.001 | 27.2 |  | 84 to 100 | 0.48±0.05 | 0.001 | 24.6 |
|  |  |  |  |  |  |  |  |  |  |
|  | *Left-Right Obliquity* | | |  |  | *Left-Right Obliquity* | | |  |
|  | *Gait Cycle %* | *DoA* | *p-value* | *max SI_norm_ %* |  | *Gait Cycle %* | *DoA* | *p-value* | *max SI_norm_ %* |
|  | 0 to 100 | 1.38±0.06 | 0.001 | 11.5 |  | 4 to 22 | 1.06±0.06° | 0.037 | 10.3 |
|  |  |  |  |  |  | 53 to 66 | 1.07±0.03° | 0.043 | 11 |
|  |  |  |  |  |  |  |  |  |  |
| *Hip* | *Flexion-Extension* | | |  |  | *Flexion-Extension* | | |  |
|  | *Gait Cycle %* | *DoA* | *p-value* | *max SI_norm_ %* |  | *Gait Cycle %* | *DoA* | *p-value* | *max SI_norm_ %* |
|  | 15 to 55 | 2.57±0.08 | 0.01 | 11.8 |  | 19 to 53 | 2.60±0.13 | 0.004 | 10 |
|  |  |  |  |  |  |  |  |  |  |
|  | *Internal-External Rotation* | | |  |  | *Internal-External Rotation* | | |  |
|  | *Gait Cycle %* | *DoA* | *p-value* | *max SI_norm_ %* |  | *Gait Cycle %* | *DoA* | *p-value* | *max SI_norm_ %* |
|  | 4 to 10 | 5.33±0.18 | 0.049 | 21 |  |  |  |  |  |
|  | 68 to 78 | 5.27±0.08 | 0.048 | 22 |  |  |  |  |  |
|  |  |  |  |  |  |  |  |  |  |
| *Knee* | *Flexion-Extension* | | |  |  | *Flexion-Extension* | | |  |
|  | *Gait Cycle %* | *DoA* | *p-value* | *max SI_norm_ %* |  | *Gait Cycle %* | *DoA* | *p-value* | *max SI_norm_ %* |
|  | 5 to 39 | 2.65±0.1 | 0.009 | 7.9 |  | 0 to 42 | 2.81±0.24 | 0.001 | 8.3 |
|  | 82 to 97 | 2.71±0.13 | 0.031 | 5.3 |  | 81 to 100 | 2.98±0.13 | 0.02 | 6.5 |
|  |  |  |  |  |  |  |  |  |  |
|  | *Internal-External Rotation* | | |  |  | *Internal-External Rotation* | | |  |
|  | *Gait Cycle %* | *DoA* | *p-value* | *max SI_norm_ %* |  | *Gait Cycle %* | *DoA* | *p-value* | *max SI_norm_ %* |
|  | 4 to 10 | 1.85±0.12 | 0.049 | 18.5 |  | 0 to 20 | 2.35±0.35 | <0.001 | 22.7 |
|  | 16 to 17 | 1.64±0.01 | 0.05 | 12.6 |  | 25 to 40 | 1.89±0.11 | 0.001 | 15.4 |
|  | 22 to 42 | 1.75±0.1 | 0.001 | 18.5 |  | 95 to 100 | 2.29±0.25 | 0.031 | 22.7 |
|  | 83 to 87 | 1.57±0.08 | 0.044 | 18.5 |  |  |  |  |  |
|  | 96 to 97 | 1.75±0.1 | 0.05 | 22.7 |  |  |  |  |  |
|  |  |  |  |  |  |  |  |  |  |
| *Ankle* | *Dorsi-Plantar Flexion* | | |  |  | *Dorsi-Plantar Flexion* | | |  |
|  | *Gait Cycle %* | *DoA* | *p-value* | *max SI_norm_ %* |  | *Gait Cycle %* | *DoA* | *p-value* | *max SI_norm_ %* |
|  |  |  |  |  |  | 12 to 13 | 1.62±0.08 | 0.05 | 8.4 |
|  |  |  |  |  |  | 35 to 37 | 1.21±0.01 | 0.05 | 6.5 |
|  |  |  |  |  |  | 45 to 48 | 1.34±0.16 | 0.039 | 7.8 |
|  |  |  |  |  |  | 79 to 94 | 1.37±0.14 | <0.001 | 8.4 |
|  |  |  |  |  |  |  |  |  |  |
|  | *Eversion-Inversion* | | |  |  | *Eversion-Inversion* | | |  |
|  | *Gait Cycle %* | *DoA* | *p-value* | *max SI_norm_ %* |  | *Gait Cycle %* | *DoA* | *p-value* | *max SI_norm_ %* |
|  | 0 to 19 | 2.34±0.18 | 0.021 | 13.4 |  |  |  |  |  |
|  | 71 to 100 | 2.6±0.22 | 0.008 | 16.4 |  |  |  |  |  |
|  |  |  |  |  |  |  |  |  |  |
|  | *Internal-External Rotation* | | |  |  | *Internal-External Rotation* | | |  |
|  | *Gait Cycle %* | *DoA* | *p-value* | *max SI_norm_ %* |  | *Gait Cycle %* | *DoA* | *p-value* | *max SI_norm_ %* |
|  |  |  |  |  |  | 92 to 98 | 1.76±0.05 | 0.034 | 24.5 |

Abbreviations: DoA, degree of asymmetry; SI_norm_, normalized symmetry index.

**Table 3.** Descriptive statistics of the maximum SI_norm_ in % and its comparison between normal and fast treadmill walking for N=60 participants.

| Segment | Motion | Treadmill Walking Normal | | | |  | Treadmill Walking Fast | | | |  |  |
| --- | --- | --- | --- | --- | --- | --- | --- | --- | --- | --- | --- | --- |
|  |  | *Mean* | *SD* | *Max* | *Min* |  | *Mean* | *SD* | *Max* | *Min* |  | *p-value* |
| *Upper Thorax* | *Flexion-Extension* | 45.56 | 12.47 | 66.67 | 12.70 |  | 44.17 | 12.61 | 65.61 | 12.64 |  | 0.201 |
|  | *Left-Right Lateral Flexion* | 13.87 | 11.07 | 60.71 | 0.56 |  | 15.36 | 12.07 | 63.82 | 0.84 |  | 0.109 |
|  | *Left-Right Rotation* | 22.28 | 14.08 | 64.70 | 0.81 |  | 22.59 | 12.30 | 58.93 | 1.28 |  | 0.765 |
|  |  |  |  |  |  |  |  |  |  |  |  |  |
| *Lower Thorax* | *Flexion-Extension* | 44.88 | 11.76 | 66.50 | 15.60 |  | 42.42 | 11.28 | 65.84 | 9.50 |  | **0.019** |
|  | *Left-Right Lateral Flexion* | 15.11 | 11.30 | 56.47 | 0.46 |  | 17.82 | 12.17 | 59.03 | 0.00 |  | **0.000** |
|  | *Left-Right Rotation* | 13.60 | 9.13 | 60.25 | 2.03 |  | 13.69 | 10.75 | 66.07 | 0.54 |  | 0.919 |
|  |  |  |  |  |  |  |  |  |  |  |  |  |
| *Upper Lumbar* | *Flexion-Extension* | 42.05 | 11.84 | 66.67 | 9.69 |  | 41.35 | 11.09 | 66.67 | 16.63 |  | 0.422 |
|  | *Left-Right Lateral Flexion* | 13.77 | 10.86 | 64.38 | 0.00 |  | 13.65 | 10.10 | 59.13 | 0.18 |  | 0.867 |
|  | *Left-Right Rotation* | 10.87 | 8.21 | 64.26 | 1.05 |  | 11.42 | 9.24 | 66.67 | 0.41 |  | 0.456 |
|  |  |  |  |  |  |  |  |  |  |  |  |  |
| *Lower Lumbar* | *Flexion-Extension* | 37.29 | 12.85 | 66.67 | 11.15 |  | 37.79 | 12.95 | 66.67 | 12.54 |  | 0.574 |
|  | *Left-Right Lateral Flexion* | 14.82 | 10.16 | 55.32 | 0.00 |  | 15.90 | 11.83 | 59.77 | 0.59 |  | 0.163 |
|  | *Left-Right Rotation* | 14.51 | 9.99 | 56.92 | 0.05 |  | 16.82 | 11.77 | 63.86 | 0.75 |  | **0.002** |
|  |  |  |  |  |  |  |  |  |  |  |  |  |
| *Pelvis* | *Posterior-Anterior Tilt* | 38.10 | 14.02 | 66.67 | 6.40 |  | 37.77 | 13.83 | 66.67 | 4.94 |  | 0.770 |
|  | *Left-Right Obliquity* | 9.83 | 10.20 | 65.57 | 0.28 |  | 10.49 | 11.56 | 62.98 | 0.05 |  | 0.474 |
|  | *Left-Right Rotation* | 12.25 | 8.51 | 50.01 | 0.67 |  | 11.88 | 8.95 | 59.82 | 0.29 |  | 0.602 |
|  |  |  |  |  |  |  |  |  |  |  |  |  |
| *Hip* | *Flexion-Extension* | 7.98 | 10.13 | 65.29 | 0.15 |  | 8.18 | 8.86 | 60.56 | 0.33 |  | 0.787 |
|  | *Adduction-Abduction* | 15.39 | 15.10 | 66.67 | 0.84 |  | 16.40 | 14.69 | 66.62 | 1.59 |  | 0.372 |
|  | *Internal-External Rotation* | 28.46 | 15.05 | 66.06 | 3.25 |  | 29.51 | 13.59 | 63.38 | 5.99 |  | 0.164 |
|  |  |  |  |  |  |  |  |  |  |  |  |  |
| *Knee* | *Flexion-Extension* | 6.62 | 6.64 | 61.39 | 0.63 |  | 7.71 | 6.19 | 62.62 | 0.40 |  | **0.045** |
|  | *Adduction-Abduction* | 29.80 | 16.00 | 66.05 | 2.38 |  | 30.33 | 13.42 | 63.55 | 2.61 |  | 0.545 |
|  | *Internal-External Rotation* | 30.06 | 13.17 | 62.85 | 6.59 |  | 27.71 | 12.44 | 62.08 | 4.34 |  | **0.009** |
|  |  |  |  |  |  |  |  |  |  |  |  |  |
| *Ankle* | *Dorsi-Plantar Flexion* | 11.77 | 9.08 | 66.67 | 0.64 |  | 10.91 | 7.88 | 58.57 | 1.045 |  | 0.294 |
|  | *Eversion-Inversion* | 21.22 | 10.39 | 57.16 | 1.87 |  | 22.97 | 11.76 | 63.11 | 1.98 |  | **0.036** |
|  | *Internal-External Rotation* | 30.00 | 14.85 | 66.61 | 1.17 |  | 31.13 | 14.18 | 66.67 | 1.22 |  | 0.261 |

Abbreviations: SD, standard deviation; Max, maximum; Min, minimum.

*Methods (Participants)*

*Was an a priori sample size estimate/power analysis performed? If so, please provide the appropriate details regarding this estimate/analysis in the manuscript. If not, please provide a rationale for not doing so in the manuscript*

Reply: We agree and thank the Reviewer for the comment. A power analysis has been included in the Methods Section accordingly to make the manuscript clearer. Based on the study results, a sample size of 60 participants with an alpha = 0.05, and a sample ratio = 1, yields a power = 0.82.

**Line 105-106**:

*A power analysis based on our study results indicates that a sample size of 60 participants with an alpha = 0.05, and a sample ratio = 1, produces a power = 0.82.*

*Figure 1 (Legend)*

*The abbreviation “L” is used twice (for “Lower” and “Lateral”). Please change one of the abbreviations to avoid confusion.*

Reply: We agree and thank the Reviewer for the comment. One of the abbreviations had been changed as suggested. The abbreviation “Lt” has been used for “Lateral”.

**Line 122-127**:

*Fig 1. Spine and lower body marker set. Prefixes denote the following: L: Left, R: Right, U: Upper, L: Lower, Lt: Lateral, and M: Medial. The following landmarks were used: Spinous process at T1 (T1), spinous process at T6 (T6), spinous process at L1(L1), spinous process at L3 (L3), spinous process at L5 (L5), thorax (TH), lumbar (LB), anterior superior iliac spine (ASI), posterior superior iliac spine (PSI), femur (THI), epicondyle of femur (KN), tibia (TB), malleoli (AK), and foot (FT).*

*Figure 1.*

*Discussion*

*- Lines 362-366, 372-373: In the clinical and research literature, the term “gait variability” refers to the stride-to-stride fluctuations in a gait parameter (e.g., step time/length, stride time/length). This is very different than the construct assessed in this study (i.e., “symmetry” as defined in the manuscript). Therefore, these sentences are not appropriate and need to be edited for clarity.*

Reply: We agree and thank the Reviewer for the comment. The sentence has been removed from the Discussion Section to increase the clarity for readership.

**Line 366-367**:

*Overall, our findings suggest that young healthy adults may be more asymmetrical during fast treadmill walking than normal treadmill walking (see Table 3).*

*- Lines 367-368: This sentence alludes to assessments made “during normal and treadmill walking”. However, no assessments were made using data collected during normal overground walking. Therefore, this sentence is not appropriate and needs to be edited for clarity.*

Reply: We agree thank the Reviewer for the comment. The sentence has been corrected in the Discussion Section accordingly to increase the clarity for readership.

**Line 367-369**:

*In addition, our results revealed standard deviations of the left side higher than the right side for hip, knee, and ankle motions during normal and fast treadmill walking.*

*- Lines 370-372: This sentence alludes to assessments made using data collected while individuals were “walking slowly”. However, no such assessments were made (asking individuals to walk at their regular walking speed is not the same as asking them to walk at a pace slower than their regular walking speed). Therefore, this sentence is not appropriate and needs to be edited for clarity.*

Reply: We agree and thank the Reviewer for the comment. We have revised the sentence accordingly in the Discussion Section to increase the clarity for readership.

**Line 371-376**:

*Even though previous reports indicate that walking slowly is more challenging to the motor control of gait than usual and faster speed walks [41, 44], differences of gait motor control between usual and faster speed walking are not clear. Therefore, the findings of this study reported as the degree of asymmetry and the normalized symmetry index may be useful indicators of the gait motor control at different walking speeds.*
